# Estimation of the stable frozen zone volume and the extent of contrast for a therapeutic substance

**DOI:** 10.1371/journal.pone.0238929

**Published:** 2020-09-17

**Authors:** Nikolai N. Korpan, Sergey G. Chefranov

**Affiliations:** 1 International Institute of Cryosurgery, Rudolfinerhaus Hospital, Vienna, Austria; 2 1st Department of Surgery, National Medical University, Kyiv, Ukraine; 3 Physics Department, Technion-Israel Institute of Technology, Haifa, Israel; 4 A.M. Obukhov Institute of Atmospheric Physics, Russian Academy of Science, Moscow, Russia; China University of Mining and Technology, CHINA

## Abstract

**Background:**

In biomedical science and clinical practice, an estimation of the stable frozen zone volume and distribution of concentration fields of injected diagnostic and healing solutions in the tissues of living organisms is of great importance and does not currently have any mathematical solution aimed at its precise evaluation.

**Objective:**

The aim of this research is the estimation of the stable frozen zone volume at ultra-low temperatures as well as the distribution of temperature areas and concentration fields of injected diagnostic and healing substances *in vitro*. The results can improve our understanding of the stable frozen zone volume and the extent of contrast for a therapeutic substance.

**Materials and methods:**

A cryogenic zone (ice ball) was generated at -180°C using liquid nitrogen without any difficulties *in vitro*. The effects of freeze-thaw processes using ultra-low temperature and the cryogenic response of a 1.5% gelatin solution in water (%g/v) kept at a constant temperature of 20°C and continuously stirred were mathematically analyzed. The stable frozen zone volume was illustrated *in vitro* and measured in terms of its length, depth and cryogenic margin using a standard medical ruler and Vernier caliper after a freezing period at -180°C, using liquid nitrogen to provide cooling and freezing of a small portion of this solution in the vessel at room temperature (20°C). Round-shaped cryoprobes with diameters of 15 mm and 50 mm were applied to create a frozen zone volume *in vitro*. A single cryoprobe was used per procedure. The sample exposure time was 3 min. After this time, the volume of the frozen region remains unchanged, which indicates that the equilibrium stationary state has been reached. The experimental design, cryogenic procedure and freeze-thaw processes of the hemisphere were described and illustrated *in vitro* item by item. The statistical analysis manifested significant differences that were found between the 50 mm and 15 mm cryoprobes with regards to the freezing diameter, depth, and cryogenic margin (*P* < 0.001).

**Results:**

An illustrated analytical mathematical solution of equations determined the stable frozen zone volume and the radius of the sphere of the frozen medium in the equilibrium stationary state. The resulting assessment provided the basis for the creation of mini- and micro-cryoprobes as well as cryoneedles for local tissue freezing in living biological structures. A solution to the equations was obtained under the boundary conditions with a set stable temperature value on the boundary surface of the cryoprobe as well as at the surface well-away from it, where the temperature is equal to the stable temperature of the environment. For example, this solution gives that in the case of a hemispherical cryoprobe radius of 1 mm, the frozen zone volume was more than three orders of magnitude greater than the volume of the cryoprobe itself and was equal to approximately 4 cm^3^. The determination of the fractal dimension can consider the individual characteristics of the spread of the contrast medium or therapeutic substance(s) in living tissue. Based on fractal theory, our innovative mathematical formulas allow for the assessment of the effective distribution of contrast medium in living biological structures, specifically for tissues assessed for diagnostic purposes, and they enable the selection of an optimal treatment strategy in medical practice.

**Conclusion:**

A simple mathematical approach to solving the problems of assessing the stable frozen zone volume and distribution of temperature areas and concentration fields of injected diagnostic and healing substances in living biological structures, particularly living tissue *in vitro*, is presented in this study. The expressed quantitative mathematical formulas determine the stable stationary frozen zone volume and provide the basis for the creation of mini- and micro-cryoprobes. The application of fractal theory is proposed for assessing the distribution efficiency of contrast medium and therapeutic substance(s) in living biological structures for diagnostic purposes and for selecting a compassionate treatment strategy in medical professional practice.

## Introduction

The problem of estimating the frozen zone volume as well as the distribution of temperature areas and concentration fields of injected diagnostic and healing solutions and impurities in the tissue of a living organism is of great practical importance for the development of cryoscience, cryomedicine and cryosurgery [1–3].

Numerous fundamental *in vitro* and *in vivo* studies have already been carried out and published in the global literature. New experimental models have been developed for the investigation of the dynamic temperature field of the frozen zone and the definition of the four phases for the effect of low temperatures on living tissue. The concepts of thermal cascade phases showing the effect of low temperatures on living tissue and the thermal synthesis of tissue response to low temperature along with its main characteristics of temperature zones resulting from low temperature exposure as theoretical models have also been previously described [1, 4].

The coldest tissue temperature is the prime factor in cell death. The changes in normal and pathological living nature, *i*.*e*., cell lines, tissue structure and fluid systems, could be dependent on different low temperature exposure, especially deep ultra-low temperatures [5–7].

In the first section, this study proposes the estimation of the stable stationary frozen zone volume and distribution of temperature areas and concentration fields of injected liquid medium reflected upon exposure to low temperatures based on the methods of mathematical physics used in the theory of turbulence and the theory of turbulent diffusion [8, 9].

As with any physical process, the effect of cold on a biological living substance and the use of low temperatures in biology and medicine can be both positive and negative. One of the basic objective principles to understand the action of ultra-low temperature *in vitro* and *in vivo* is estimating the frozen zone volume (FZV).

Moreover, considering the complexity in the formulation of the corresponding boundary value problem, which provides mathematical modeling of these processes, it is of interest to consider a simplified formulation for the spherically symmetric case of the stable stationary temperature distribution.

The second section proposes the application of fractal theory to assess the effective measurement of the spread of contrast medium in tissue [10].

The authors’ basic theoretical, experimental and mathematical research has provided further scientific and practical foundations on the unexpected role of modern cryoscience, cryobiology, cryomedicine, cryosurgery and cryotechnology in different areas of biomedical science and practical medicine, especially surgical oncology and biogenic cryoimmunology.

A simple mathematical approach to solving the problems of assessing the stable frozen zone volume and distribution of temperature areas and concentration fields of injected diagnostic and healing substances in living biological structures, particularly living tissue *in vitro*, is presented in this study.

## Materials and methods

In this paper, we used experimental observations of the effects of freeze-thaw processes using ultra-low temperatures in terms of cryoprobes of different sizes and shapes. The investigations were carried out by means of two selected disc-shaped cryoprobes with diameters of 15 mm and 50 mm. A single cryoprobe was utilized per procedure. A hemispheric cryogenic zone (ice ball) was generated at -180°C using liquid nitrogen without any difficulties *in vitro*. The simple exposure time was 3 min. After this time, a stable stationary frozen volume is always found *in vitro*.

**Fig 1:**
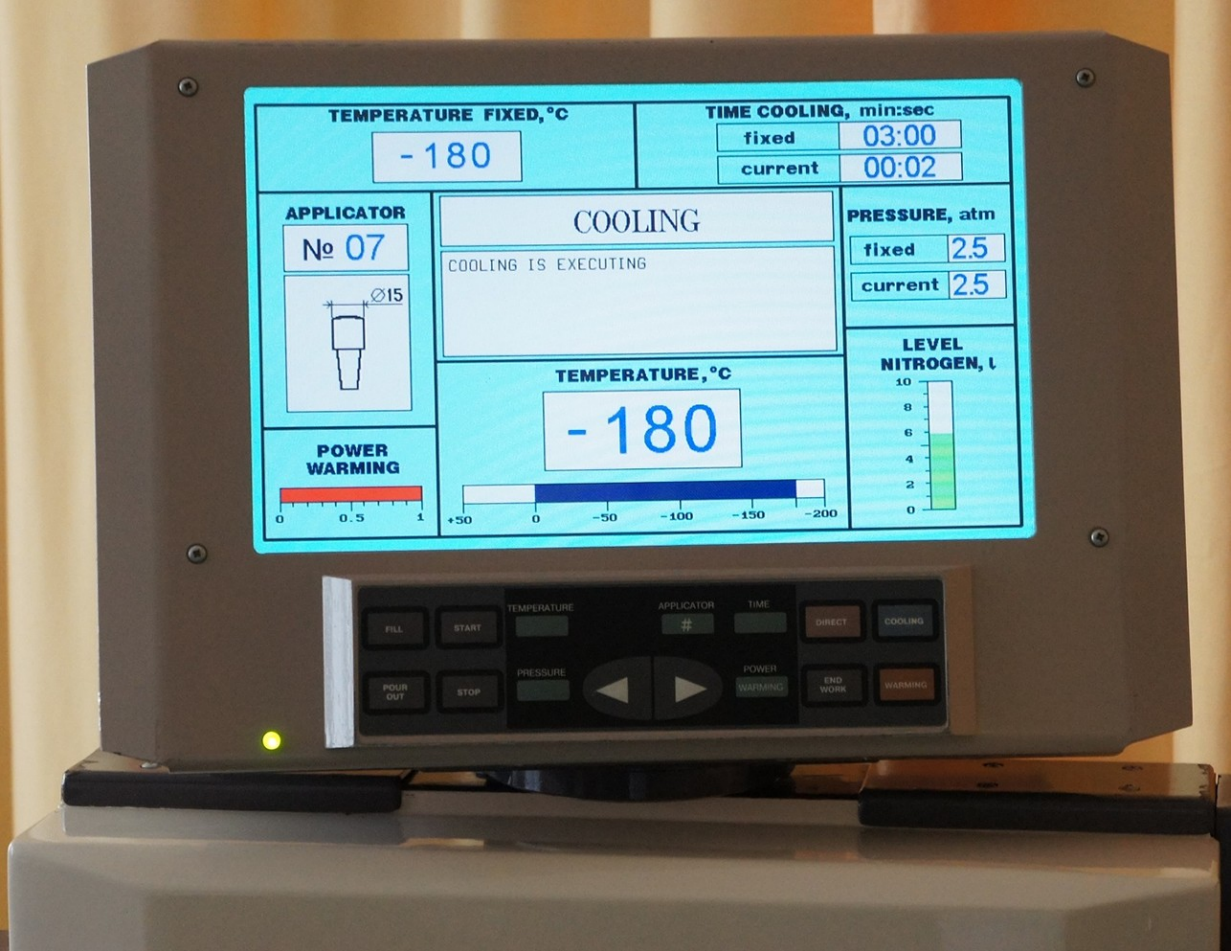
Mobile universal cryogenic complex (UCC) based on liquid nitrogen as the refrigerant produced by CryoPulse Co., Kyiv, Ukraine. View of electronic device display.

An innovative mobile universal cryogenic complex (UCC) was applied for the creation of a cryohemispheric formation of a stable frozen zone volume (Fig 1).

The *in vitro* and *in vivo* methods used in the presented mathematical study have been previously extensively described and published in professionally qualified literature [5, 11].

We used the measurement of a formatted stable frozen zone volume (ice ball) in terms of the length and depth as well as cryogenic margin using a standard medical ruler and Vernier caliper after a freezing period at -180°C, which is decidedly illustrated in this study.

The effect of freeze-thaw processes using ultra-low temperature and the cryosurgical response of a 1.5% solution of gelatin in water (%g/v) were mathematically analyzed to achieve the objective of this study. The experimental design, operative procedure, cryosurgical approach and freeze-thaw processes of *in vitro* and *in vivo* hemispheric cryoprobes with diameters of 5 mm to 50 mm have already been described and illustrated in great detail [1, 3].

Experimental and clinical investigations *in vivo* paved the way for a mathematical estimation of the frozen zone volume (FZV) and the extent of contrast substance distribution in living biological matter [5, 7, 12].

The simple mathematical approach was undertaken to solve the problems of assessing the stable stationary frozen zone volume and distribution of temperature areas as well as concentration fields of injected diagnostic and healing substance(s) in living biological structures *in vitro* and *in vivo*. To determine the effective measurement of the distribution of contrast agents *in vivo*, the concept of the fractal dimension of the region, in which these substances are observed, is used [8, 13].

We used a stationary solution of the heat equation for the case of spherical symmetry under given boundary conditions to analyze these data [10].

### Statistical analysis

The statistical analysis was performed with IBM^®^ SPSS^®^ Statistics (Version 26). One-way analysis of variance (ANOVA) was performed followed by simple contrasts comparing the 50 mm cryoprobe to the 15 mm cryoprobe. Results were reported as the mean ± SD. Mean differences associated with *P* values less than 0.05 were considered statistically significant. Welch’s correction was applied for heterogeneous variances. Significant differences were found between the 50 mm and 15 mm probes with regards to the freezing diameter, depth, and cryogenic margin (*P* < 0.001).

## Results

### Section I. Determination of the stable frozen area volume

#### 1A

In conjunction with the development of cryosurgery methods, there is a need to determine quantitative parameters characterizing the stable stationary volume of frozen tissue in contact with the cryoprobe, in which liquid nitrogen is circulated and the temperature of the liquid reagent is maintained.

**Fig 2:**
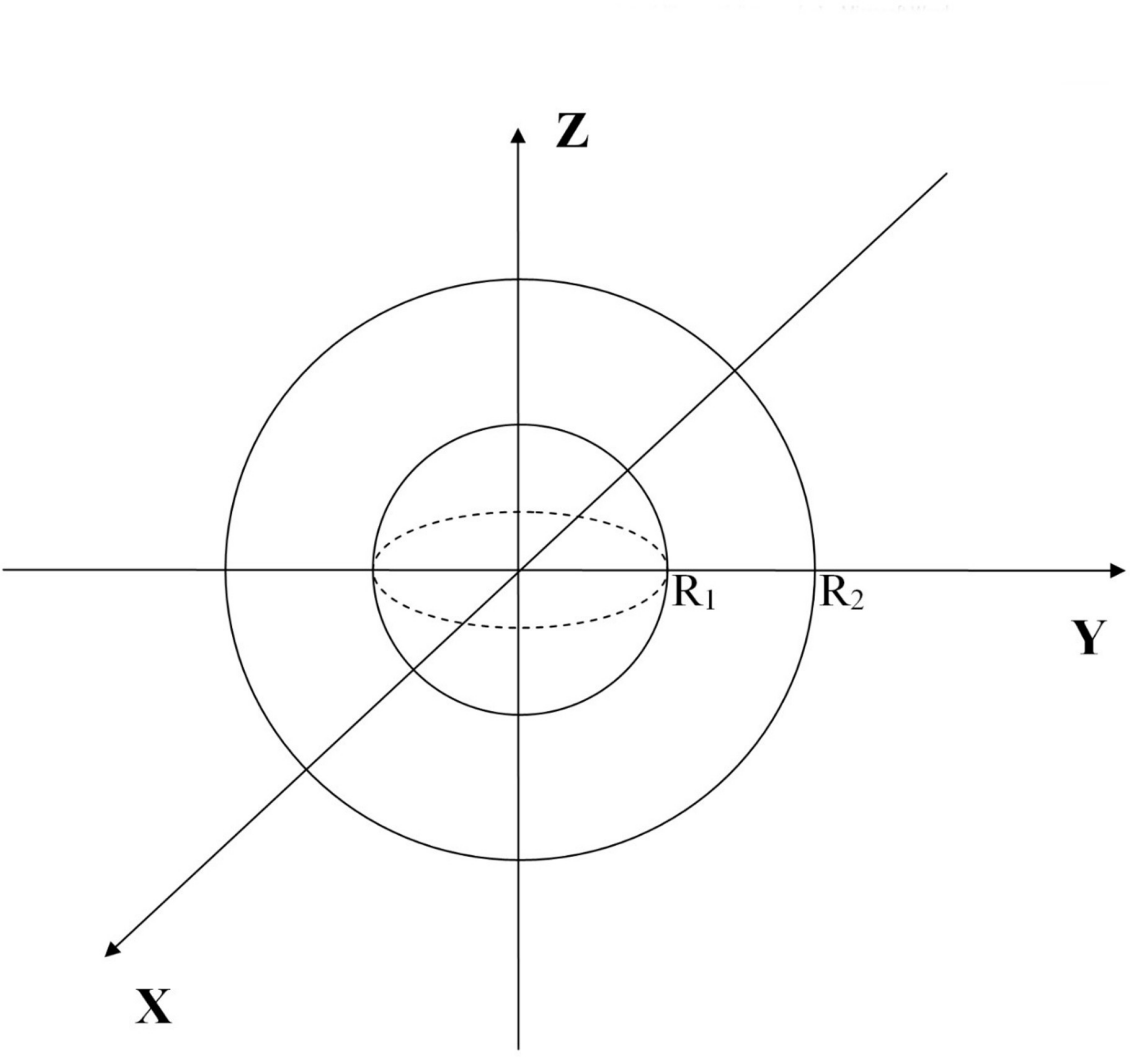
The inner sphere of radius *R*_1_ corresponds to a cryoprobe surface having a predetermined temperature *T*_1_. The outer sphere of radius *R*_2_ defines the surface inside the medium on which a constant temperature *T*_2_ is maintained, as determined by the equilibrium state of the heat balance with the environment, an unlimited heat reservoir in the case of a non-living environment surrounding the applicator and a condition of homeostasis in the case of an environment in a living organism.

Currently, cryoprobes with various contact surface shapes are commonly used [10, 13, 14]. However, for simplicity, we will assume a sphere of radius *R*_1_ having a temperature *T*_1_ = 93^0^*K*, which has a center coinciding with the center of a sphere of radius *R*_2_, assuming that room temperature *T*_2_ = 293^0^*K* is maintained on the surface of the outer sphere of radius *R*_2_ (Fig 2).

It is necessary to find the volume of the medium bounded by a spherical surface until the radius *R*_*f*_ has a freezing point of water *T*_*f*_ = 273^0^*K* (hereinafter, the temperature is measured in Kelvin).

To implement this procedure, we consider a heat equation in the spherical coordinate system written for the spherically symmetric case when the temperature distribution *T* does not depend on spherical angular variables *θ*,*φ* and is determined only by the radial coordinate *r*, measured from the origin coinciding with the center of the cryoprobe sphere. The equation has the following form [8–10, 13, 14]:

$$\frac{\partial T}{\partial t}=\chi \Delta T+Q;\Delta =\frac{1}{r^{2}}\frac{\partial }{\partial r}r^{2}\frac{\partial }{\partial r}$$ (1)

In (1), the left side has a partial derivative with respect to time *t*, and the right side has a Δ denoting the Laplace operator in the spherically symmetric case when the field of the temperature distribution is assumed to be independent of spherical angular coordinates *θ*,*φ*. At the same time, *χ* denotes the coefficient of thermal diffusion, which for water depends on the temperature (at room temperature for clean water *χ*≅1.42×10^−3^*cm*^2^/sec;*T* = 293^0^*K*). Value *Q* is determined by the power of internal heat sources, for example, when the tissue of a living organism is considered a medium. In addition, function *Q* must also take into account the heat release that occurs during the phase transition of water into ice, when 334.7 *J* is released during the transition of one gram of water into ice.

From the observed data, it follows that the limiting volume of the frozen area is reached in approximately three minutes. In this regard, instead of the nonstationary Eq (1), one can use the corresponding stationary equation:

$$\begin{array}{l} \Delta T+\frac{1}{\chi }Q=0;\\ Q=\lim_{t\to t_{eq}}(Q_{in}(r,t)-Q_{out}(r,t)) \end{array}$$

In the general case, with the known form of the functions of heat release *Q*_*in*_ and heat removal *Q*_*out*_, it is possible, using numerical methods, to obtain a solution to this equation and determine the distribution of the temperature field. From this temperature distribution, the volume of the frozen region, which corresponds to the isotherm of the freezing point of water at 273 Kelvin (zero degrees centigrade), can be established. Instead, for simplicity, we consider only the assumption of complete compensation of the processes of heat release and heat removal when the balance of these processes and the value of *Q* = 0 in (1) and the corresponding stationary equation in the limit $\frac{\partial T}{\partial t}\to 0$ are established at a finite time *t* = *t*_*eq*_.

For simplicity, instead of the non-stationary complex process of forming a frozen sphere, the resulting equilibrium temperature distribution and the corresponding limit volume of the frozen region should be considered. In this regard, it is sufficient to consider the stationary solution of the heat conduction Eq (1), which takes into account the presence of a balance between the inflow and outflow of heat, leading to the stationary equation Δ*T* = 0, when $\frac{\partial T}{\partial t}-Q\to 0$ in (1). In this case, the characteristic time *t*_*eq*_ for establishing the equilibrium temperature distribution is determined by the values of the medium's thermal conductivity coefficient *χ*, the size of the frozen region *R*_*f*_, and the rate of the heat removal process $\tau_{Q}^{-1}$ along with it’s inflow $\tau_{T}^{-1}$ under specified stationary boundary conditions for the temperature field. In all experiments, the time to establish the equilibrium volume of the frozen region has a value *t*_*eq*_≈1.8×10^2^ sec regardless of the size of the cryoprobe used. However, there is no significant change in the volume of the frozen area for *t*>*t*_*eq*_.

Thus, in the stationary limit for *t*>*t*_*eq*_, instead of Eq (1), the Laplace equation Δ*T* = 0 needs to be solved under the following boundary conditions:

$$\begin{array}{l} T=T_{1},r=R_{1};\\ T=T_{2},r=R_{2} \end{array}$$ (2)

The solution of equation Δ*T* = 0 under boundary conditions (2) is obtained in the analytical form without any dependence on the thermal conductivity coefficient:

$$T=C_{1}-\frac{C_{2}}{r};C_{1}=\frac{\gamma T_{2}-T_{1}}{\gamma -1};C_{2}=R_{1}\frac{\gamma (T_{2}-T_{1})}{\gamma -1};\gamma =\frac{R_{2}}{R_{1}}>1$$ (3)

In view of (3), it is possible to determine the radius of the sphere in the frozen medium, which has the following representation:

$$R_{f}=R_{1}\frac{\gamma (T_{2}-T_{1})}{\gamma (T_{2}-T_{f})+T_{f}-T_{1}}$$ (4)

For the cryoprobe temperature noted above, *T*_1_ = 93^0^*K*, the external temperature *T*_2_ = 293^0^*K* and the freezing temperature *T*_*f*_ = 273^0^*K*, expression (4) is converted to the following form:

$$R_{f}=R_{1}\frac{10\gamma }{\left(\gamma +9\right)}$$ (5)

In the limit, when the radius of the cryoprobe *R*_1_ is much smaller than the radius *R*_2_ and when $\gamma =\frac{R_{2}}{R_{1}}>>1$, then the following formulas can be derived from (4) and (5), respectively:

$$R_{f}=R_{1}\frac{\left(T_{2}-T_{1}\right)}{\left(T_{2}-T_{f}\right)}$$ (6)

$$R_{f}=10R_{1}$$ (7)

Therefore, for example, from (7), it already follows that regardless of the parameter $\gamma =\frac{R_{2}}{R_{1}}$, the ratio of the radius of the frozen region to the radius of the cryoprobe is 10, and accordingly, the volume of the frozen zone is approximately 1000 times the volume of the cryoprobe in the case of the considered temperatures (when from (6), it follows (7)). For example, for mini-cryoprobes and/or micro-cryoneedles having a spherical shape and a radius of 0.1 cm, the volume of the frozen area will be approximately equal to 4 cm^3^.

The resulting assessment provides the basis for the development of mini- and micro-cryoprobes and/or cryoneedles for tissue freezing in internal areas well below the body surface.

Formulas (4)-(7) obtained for the spherical surface of the cryoprobe, taking into account the application of the principle of symmetry, can also be used for a hemispherical cryoprobe for arbitrary values of the parameter *γ*.

#### 1B

**Fig 3:**
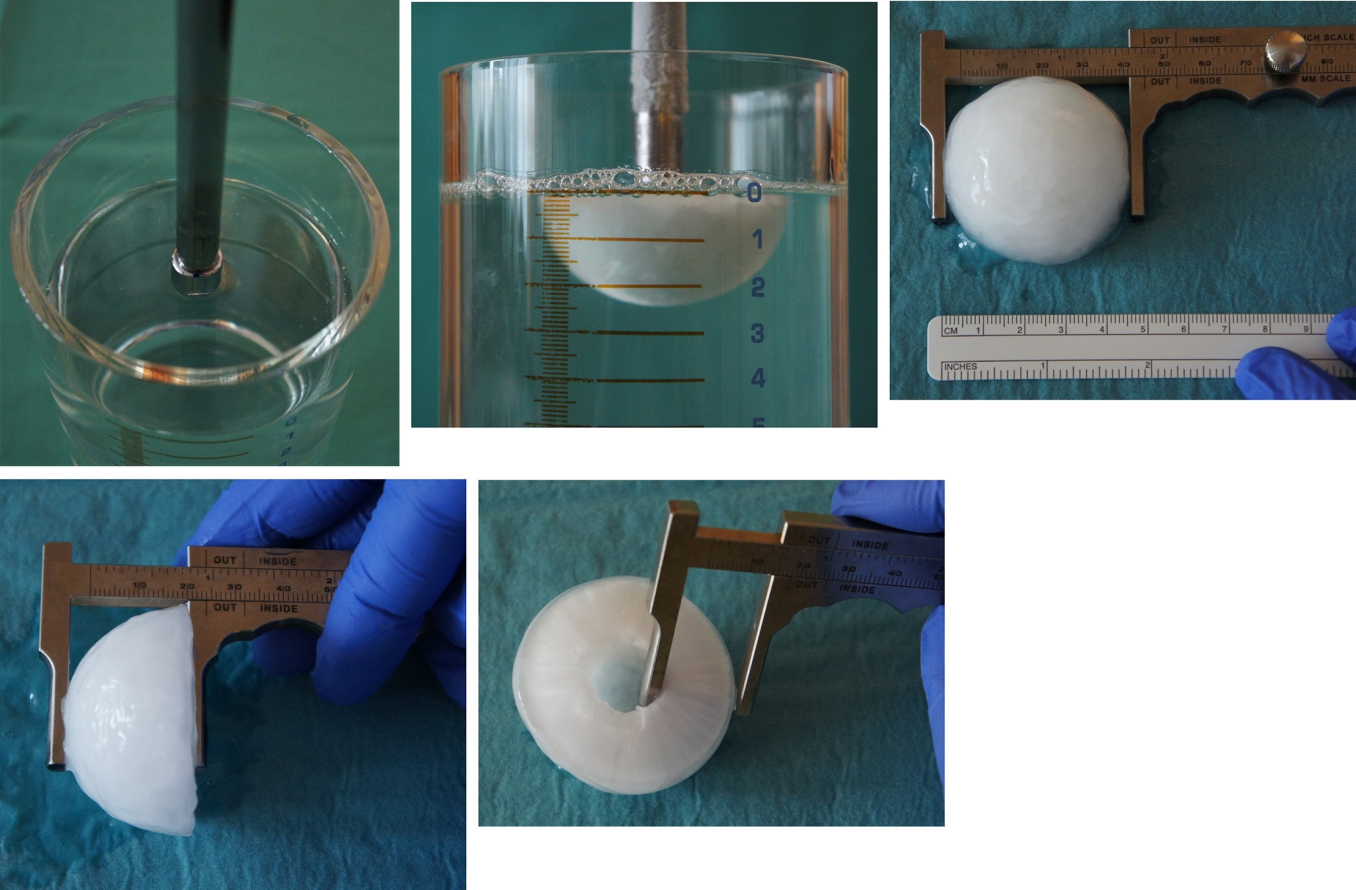
A-E Cryohemispheric formation using a cryoprobe 15 mm in diameter at -180°C: freezing diameter 45.6±0.3 mm, depth 24.4±0.5 mm and cryogenic margin 18.3±0.3 mm after a freezing duration of 3 min. (A) start; (B) after 3 min; (C) width of the cryogenic zone; (D) depth of the cryogenic zone; (E) margin of the cryogenic zone. The part of the cryoprobe immersed in the medium in Fig 3A is modeled using the bottom hemispheres, i.e., located at Z<0, of radius *R*_1_ (Fig 2). The frozen zone volume (Fig 3B) is mathematically modeled by a hemisphere of radius *R*_*f*_, whose value is determined according to formula (5).

Let us compare formula (5) with experimental data from observations of the effects of freezing close to living biological structures shown in Fig 3A–3E.

Based on (5) and the experimental data mentioned above, the value of radius *R*_2_≈28.9*mm* (because in (5) *γ*≈3.857 for this data) of the outer sphere can be determined when room temperature is maintained.

As in the previous case, it is possible based on (5) to obtain the estimations *γ* = 1.514;*R*_2_ = 37.85*mm*. Thus in this case we have *R*_2_−*R*_f_≈1.85*mm*, while in the case of the cryoprobe in Fig 3, this difference is larger and equal to *R*_2_−*R*_*f*_≈6.4*mm*. For a smaller cryoprobe this difference only grows. This is important in applications for choosing the radius of the cryoprobe.

Considering the case corresponding to parameter value *γ* = 1.14, the radius of the frozen hemisphere is equal to *R*_*f*_≈27.5*mm* when *R*_1_ = 25*mm* according to (5).

Additionally, in this case, the deviation from sphericity may be due to the relatively more noticeable effect of the flat border on the parts of the freezing zone close to it. This accordingly leads to a slight deviation from purely spherical symmetry. For small cryoprobe sizes, this deviation from sphericity no longer appears.

#### 1C

**Fig 4:**
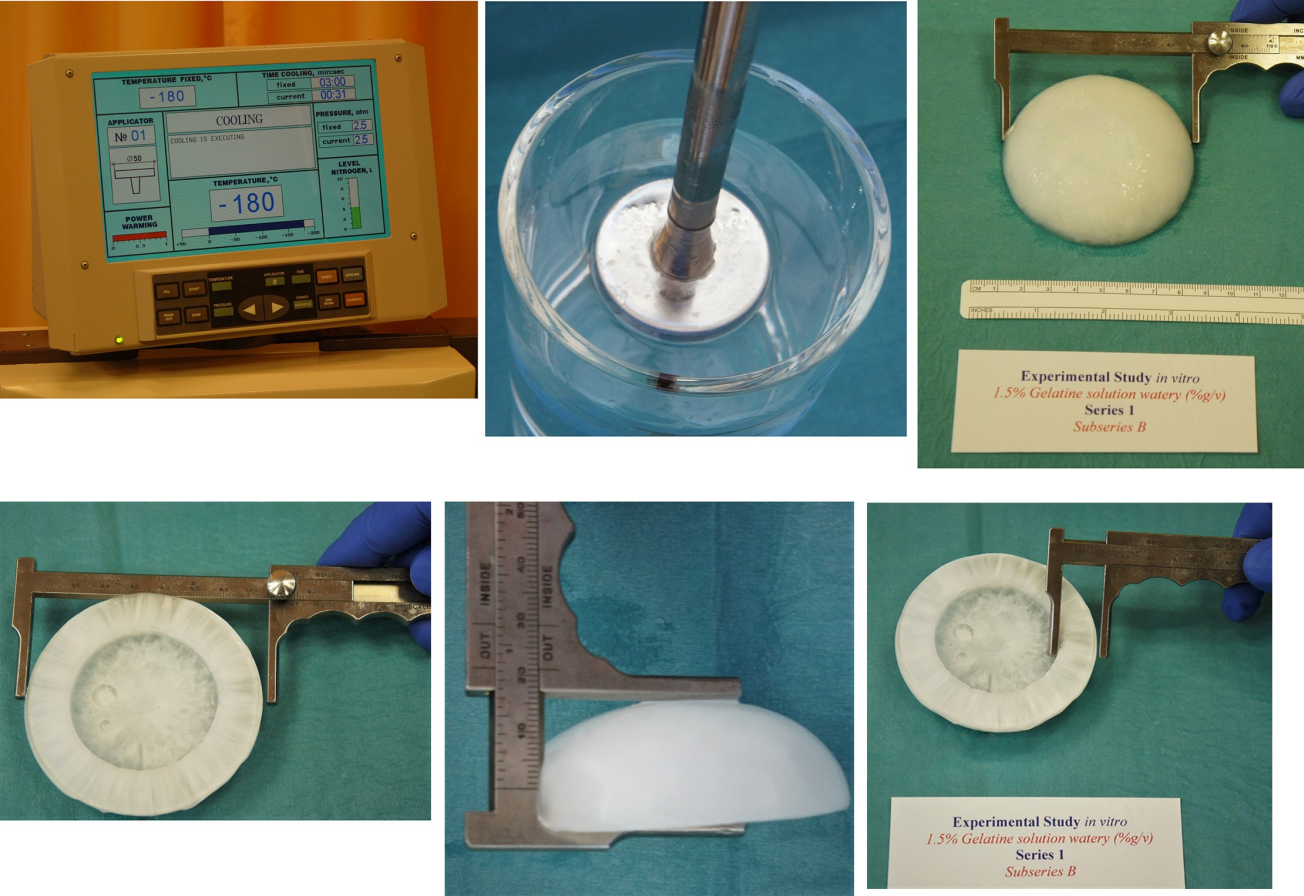
A-F Cryohemispheric formation using a cryoprobe 50 mm in diameter at -180°C: freezing diameter 72.7±0.7 mm, depth 28.6±1.0 mm and cryogenic margin 20.5±0.9 mm after a freezing duration of 3 min. (A) exposure time at -180°C using a cryoprobe 50 mm in diameter; (B) start; (C,D) the measurement of the stable formatted frozen zone volume (ice ball) is illustrated after freezing for 3 min at -180°C by utilizing a Vernier caliper to determine the length; (E) depth; and (F) cryogenic margin.

**Fig 5:**
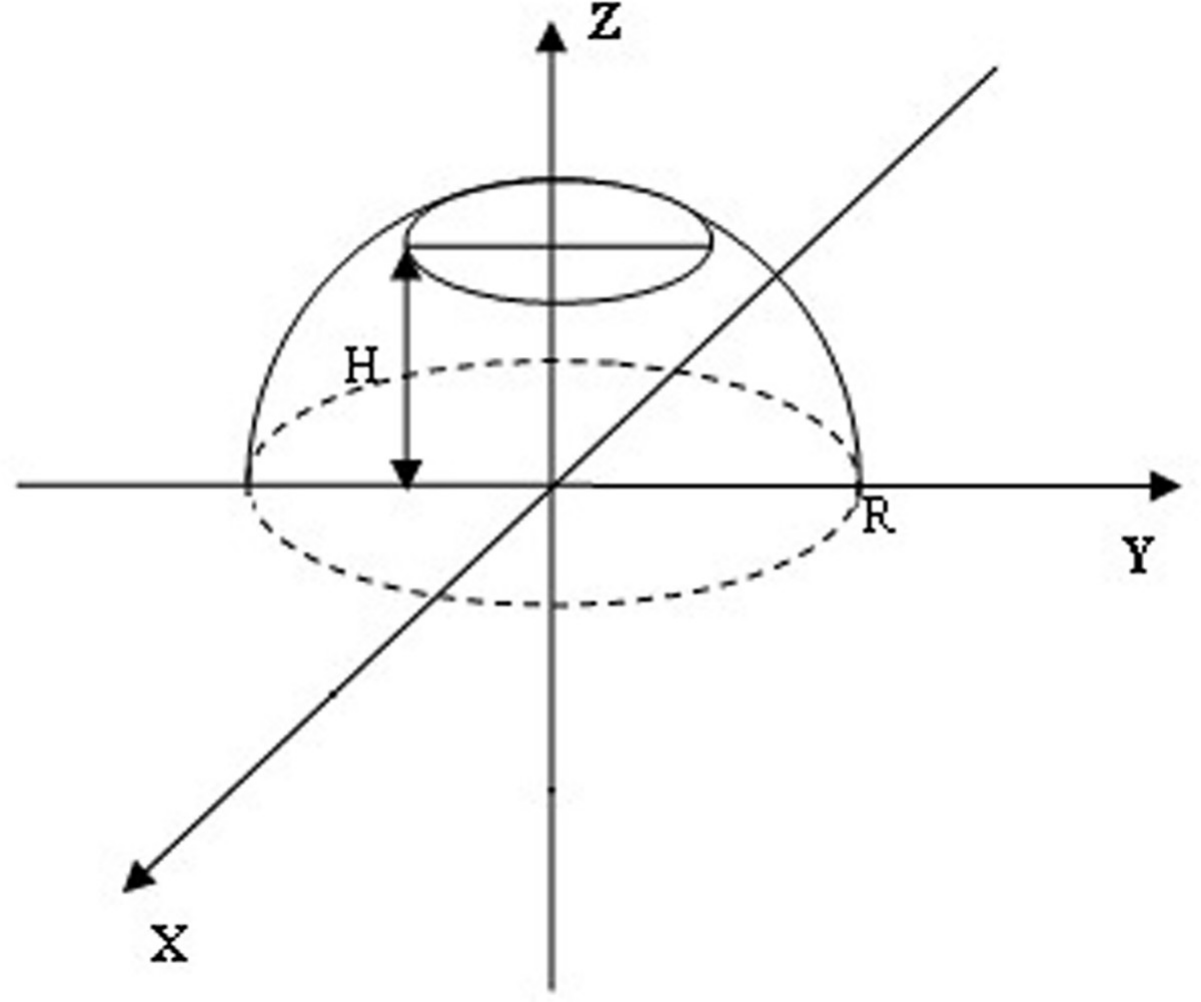
Truncated hemispheric radius R, modeling the frozen zone volume from the flat cryoprobe, located in the x-y plane. Here, the value H determines the depth to which the medium freezes in the direction of the z-axis.

To estimate the volume of the frozen zone shown in Fig 4B, we model it in the form of a truncated sphere, as shown in Fig 5.

The volume of the figure depicted in Fig 5 has a form according to the corresponding volume integral:

$$V=\frac{\pi R^{2}H}{3}(3-\frac{H^{2}}{R^{2}})$$ (8)

Using (8), we can estimate the volume of the frozen zone shown in Fig 4B, which for the dimensions shown there is equal to *V*≈50.7 cm^3^.

### Section II. Determination of the measurement of the distribution area of diagnostic contrast medium and therapeutic substance(s)

Let us now consider the task of determining the effective cross-sectional area of a tissue volume in which a given volume of contrast medium or therapeutic substance(s) is distributed. For practical purposes, the inverse problem of establishing the necessary volume of an injected contrast medium or therapeutic substance(s) to cover a given cross-sectional area of the investigated tissue volume is also important.

The distribution of contrast medium and therapeutic substance(s) in a living organism, in contrast to the distribution of temperature fields, is heterogeneous, and it is not only diffuse inside the tissue fluid but also a complex branched system of blood and lymph vessels and capillaries form a fractal-type structure characterized by its large-scale invariance.

In this regard, to solve the indicated direct and inverse problems, it seems possible to use the methods of fractal theory developed for structurally complex turbulent flows of liquid and gas (8). Moreover, the concept of fractal flow is associated with a difference in the effective dimension of the corresponding structures compared to their physical (or topological) dimension. For example, the surface separating regions with finite and zero vorticity can be so complex that their effective dimension may no longer be an integer. At the same time, the shape exceeds the usual two-dimensional dimension characterizing such surfaces locally, but it is not equal to the three dimensions characterizing a homogeneous volume structure.

Similarly, when the contrast medium or therapeutic substance(s) spreads in living tissue, the dimension of the area occupied by this substance in any flat (having a two-dimensional dimension) section of the tissue volume is a complex heterogeneous structure having a nonintegral fractal dimension 1 <d <2, which can be determined as follows [10]:

$$d=\lim_{\varepsilon \to 0}(\ln N(\varepsilon )/\ln(1/\varepsilon ))$$ (9)

where *N*(*ε*) = the number of sites with a transverse length covering the considered surface on which the contrast medium or therapeutic substance(s) is distributed in the limit when the size *ε*→0. It should be noted that due to the inhomogeneous distribution of the contrast medium or therapeutic substance(s) in the tissue volume, the dimension of the region occupied by the contrast medium or therapeutic substance(s) will no longer be three-dimensional but will have a dimension of 2 <d <3. Accordingly, a flat section of such a fractal region occupied by a contrast medium will have the indicated dimension 1 <d <2.

At the same time, analogous with the theory of turbulence, the introduction of the minimum and maximum scales *η* and L is permissible; accordingly, for scales smaller than the specified minimum scale and scales larger than the maximum scale, the distribution of contrast medium and therapeutic substance(s) in the tissue can already be considered uniform. In these areas, the usual relationship between the volume occupied by the contrast medium or therapeutic substance(s) should be V, and the cross-sectional area should be S, which has the following form:

$$V=CS^{3/2}$$ (10)

In Eq (10), the value of C is a constant coefficient of proportionality, which can be determined from empirical data. For example, from the available data on the distribution of a contrast medium, it follows that C = 0.001.

**Fig 6:**
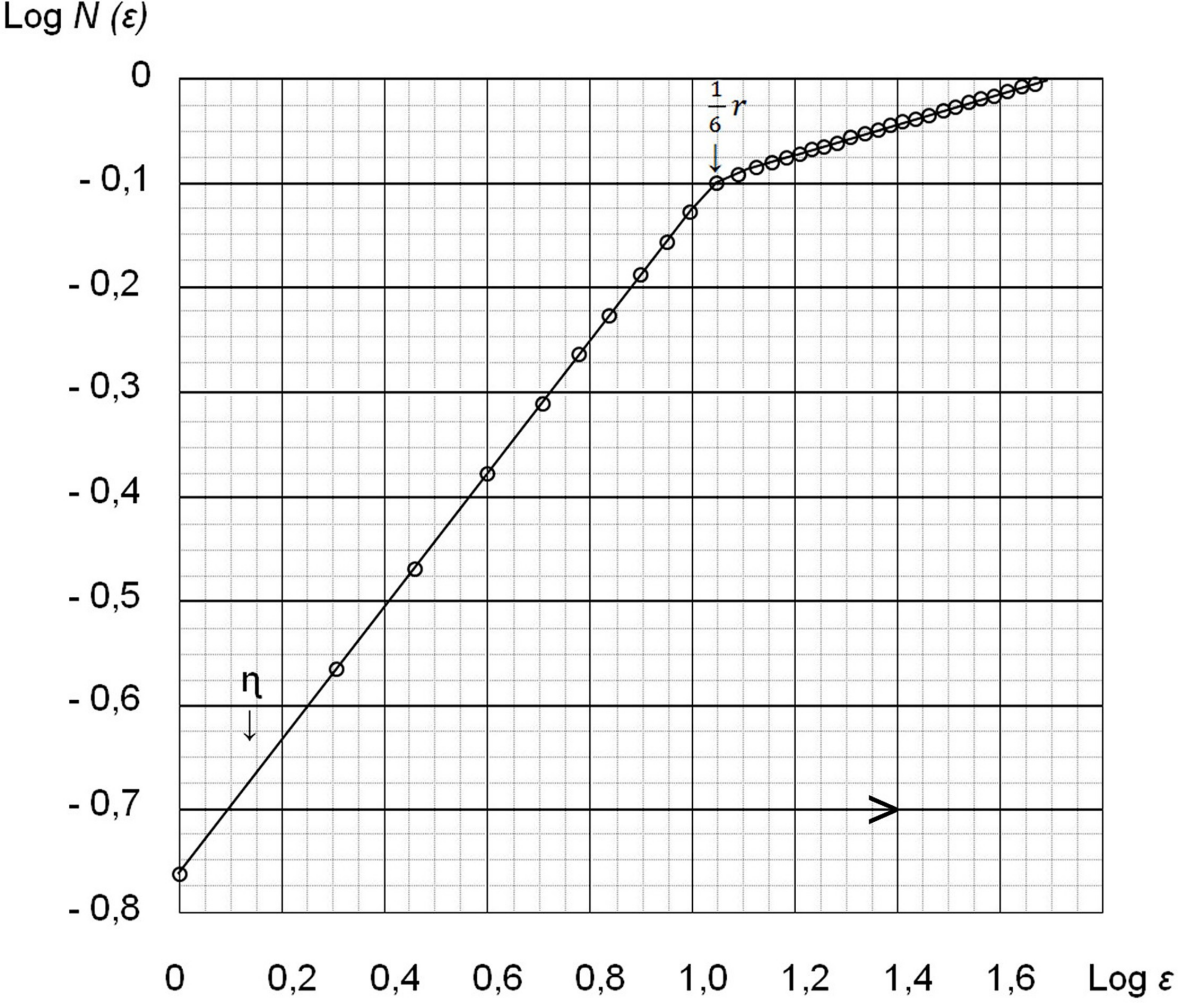
Schedule *N*(*ε*) for the cross-section of the turbulent volume. Boundary layer.

A more accurate calculation, taking into account scales larger than *η* and smaller than L, requires additional research similar to that shown in Fig 6 for the turbulent boundary layer, from which it will be possible to determine the fractal dimension of this region of intermediate scales, in which the contrast medium or therapeutic substance(s) has spread. In this case, the general formula, from which it is possible to determine the necessary volume of contrast medium or therapeutic substance(s) introduced, will have the following form:

$$V=\sum_{l<\eta }C_{\eta }S_{\eta }^{3/2}+\sum_{L<l}C_{L}S_{L}^{3/2}+\sum_{\eta <l<L}C_{f}S^{3/d}$$ (11)

Thus, a preliminary determination of the fractal dimension d is necessary, which can take into account the individual characteristics of the spread of contrast medium or therapeutic substance(s) in living biological tissue. On this basis, it is possible to solve the problems formulated above.

At the same time, for a rough estimate of the volume of injected contrast medium or therapeutic substance(s), formula (10) can be used, which accounts for only the second term on the right-hand side of (11).

## Discussion

A simple useful approach to solving the problems of assessing a stable stationary frozen region and the distribution of temperature fields in living biological structures, particularly living tissue *in vitro*, is presented. The advantage of the proposed solution, which is consistent with observational data, is the universality of the result, which can be used to estimate the size of the frozen tissue region *in vivo*. The disadvantage in this case is the inability, within the framework of this approach, to estimate the time to establish the equilibrium temperature distribution *t*_*eq*_, which can be determined only when solving the complete nonstationary problem of the propagation of the freezing area.

The mathematical formulas for calculating a passive admixture, whether a contrast medium or therapeutic substance(s), presented in this scientific study can serve as a theoretical basis for the use of more accurate volumes of contrast agents and their solutions for diagnostic purposes as well as therapeutic substance(s) in practical medicine. The volumes of various diagnostic substances introduced into the human body of a healthy and sick person, especially contrast and radioactive substances and their solutions for the purpose of diagnosis, as well as the parenteral administration of drugs, especially cytostatics, are not harmless and in many cases cause side effects of varying severity. Among these side effects, systemic and local-regional allergic and endotoxic reactions of mild to severe manifestations have been observed [15].

The conducted research opens up a completely new area in the practical application of cytostatics in oncology to use both systemic and local-regional chemo- and immunotherapy, including anticancer treatment options. In this case, the calculated dose of the administered chemotherapeutic agents, which have a toxic effect and are accompanied by side effects, is of particular importance for each cancer patient who has a weakening of the immunological protective mechanisms of the body, expressed to a varying degree of severity [16].

Additionally, the discussion presented in this paper can be generalized to consider the nonstationary processes realized during the propagation of temperature fields in living matter, in particular, the human body in the presence of internal heat sources that support homeostasis [17–19].

It is also of interest to further develop the proposed approach for already dynamically active impurities, in particular, in connection with the possibility of mathematical modeling of threshold processes of carcinogenesis based on the analogy with threshold phenomena in hydrodynamics [3, 11, 20–22].

## Summary

The presented and illustrated quantitative mathematical estimates provide the basis for the creation of mini- and micro-cryoprobes and cryoneedles that can provide a given frozen zone volume in living tissue not only near the surface of the body but also in areas well below the surface of the body. For example, if the radius of the hemispherical cryoprobe is 1 mm, the frozen zone volume can be a thousand times greater than the volume of the cryoprobe itself and can be approximately 4 cm^3^.

The analytical dependence of the stable frozen zone volume was obtained on the parameters of the cryogenic applicator (cryoprobe) as well as on the initial temperature and the thermal conductivity of the tissues in living biological structures, which are exposed to ultra-low temperatures locally.

The application of fractal theory for assessing the distribution efficiency of contrast medium and therapeutic substance(s) in living biological structures, in particular biological tissues, is proposed, which is necessary for diagnostic purposes and choosing a strategy with the gentlest treatment.

The presented basic theoretical, experimental and mathematical research has provided further scientific and practical foundations on the unexpected roles of modern cryoscience, cryobiology, cryomedicine, cryosurgery and cryotechnology in different areas of biomedical science and practical medicine, especially surgical oncology and biogenic cryoimmunology.
